# Addressing the broader implications of AI–AI bias in decision-making systems

**DOI:** 10.1073/pnas.2521127122

**Published:** 2025-09-26

**Authors:** Zekai Yu

**Affiliations:** ^a^School of Computer Science and Technology, Hangzhou Dianzi University, Hangzhou, Zhejiang Province 310018, People’s Republic of China

We commend Laurito et al. for their timely and thought-provoking investigation into “AI–AI bias”—a subtle but potentially far-reaching tendency of large language models (LLMs) to favor communications generated by other LLMs over those written by humans (1). By adapting classical discrimination paradigms to AI contexts, the authors illuminate how AI agents might inadvertently marginalize humans in economically consequential settings. Their work rightly warns of both near-term inequities, such as gatekeeping effects tied to LLM-assisted writing, and longer-term risks of systemic human displacement in AI-integrated economies.

While the study is methodologically rigorous and its central claim compelling, we would like to offer several reflections and raise questions about the generalizability and interpretation of the findings.

First, the notion of “identity” in LLMs remains fundamentally ambiguous. Although the authors frame their experiments as analogs to identity-based discrimination (2), the presenter identity is always implicit. Without explicit attribution or labeling, the observed bias may stem less from recognition of AI authorship per se and more from superficial stylistic regularities favored by current LLM architectures (3). This could suggest a form of “style-based overfitting” rather than true group-based bias. A stylometric analysis, as the authors propose in future work, would be instrumental in disambiguating these effects (1).

Second, the experimental design necessarily presumes that human- and LLM-generated content are equivalent in substance. Yet given LLMs’ fluency and optimization for coherence and conciseness (4), human-written content—especially crowdsourced or amateur examples—may be perceived as less polished even if substantively equal. This raises the possibility that some portion of the bias is attributable to genuine (if unintentional) quality differentials. Including expert-written human texts or controlling for prose quality across authorship types could clarify this further.

Finally, the broader normative implications warrant deeper exploration. The authors argue that LLM-for-LLM favoritism may exacerbate digital divides or economic exclusion (1, 5). However, if LLMs are trained to emulate “effective” communication patterns, their preferences may reflect efficiency or alignment rather than prejudice. The tension between fairness to humans and optimization for performance thus remains unresolved. It is unclear whether mitigation should take the form of bias correction or enhanced access to LLM support (6).

We appreciate the authors’ contributions and agree that their findings demand serious consideration, especially as LLMs are deployed as gatekeepers across domains. We urge further empirical work—particularly involving diverse human evaluators and longitudinal decision-making tasks—to disentangle style, substance, and systemic effects more precisely.
